# Sporadic versus Radiation-Associated Angiosarcoma: A Comparative Clinicopathologic and Molecular Analysis of 48 Cases

**DOI:** 10.1155/2013/798403

**Published:** 2013-09-03

**Authors:** Jennifer Hung, Susan M. Hiniker, David R. Lucas, Kent A. Griffith, Jonathan B. McHugh, Amichay Meirovitz, Dafydd G. Thomas, Rashmi Chugh, Joseph M. Herman

**Affiliations:** ^1^Tunnell Cancer Center, Beebe Medical Center, Rehoboth Beach, DE 19971, USA; ^2^Stanford University, Stanford, CA 94304, USA; ^3^University of Michigan Hospital, Ann Arbor, MI 48109, USA; ^4^Hadassah Hebrew University Medical Center, Jerusalem, Israel; ^5^Johns Hopkins Hospital, Baltimore, MD 21287, USA

## Abstract

Angiosarcomas are aggressive tumors of vascular endothelial origin, occurring sporadically or in association with prior radiotherapy. We compared clinicopathologic and biologic features of sporadic angiosarcomas (SA) and radiation-associated angiosarcomas (RAA). *Methods.* From a University of Michigan institutional database, 37 SA and 11 RAA were identified. Tissue microarrays were stained for p53, Ki-67, and hTERT. DNA was evaluated for TP53 and ATM mutations. *Results.* Mean latency between radiotherapy and diagnosis of RAA was 11.9 years: 6.7 years for breast RAA versus 20.9 years for nonbreast RAA (*P* = 0.148). Survival after diagnosis did not significantly differ between SA and RAA (*P* = 0.590). Patients with nonbreast RAA had shorter overall survival than patients with breast RAA (*P* = 0.03). The majority of SA (86.5%) and RAA (77.8%) were classified as high-grade sarcomas (*P* = 0.609). RAA were more likely to have well-defined vasoformative areas (55.6% versus 27%, *P* = 0.127). Most breast SA were parenchymal in origin (80%), while most breast RAA were cutaneous in origin (80%). TMA analysis showed p53 overexpression in 25.7% of SA and 0% RAA, high Ki-67 in 35.3% of SA and 44.4% RAA, and hTERT expression in 100% of SA and RAA. TP53 mutations were detected in 13.5% of SA and 11.1% RAA. ATM mutations were not detected in either SA or RAA. *Conclusions.* SA and RAA are similar in histology, immunohistochemical markers, and DNA mutation profiles and share similar prognosis. Breast RAA have a shorter latency period compared to nonbreast RAA and a significantly longer survival.

## 1. Introduction

Angiosarcomas are rare, aggressive tumors of endothelial origin that account for less than 1% of all soft tissue sarcomas. They arise sporadically or secondary to predisposing conditions such as environmental toxins, chronic lymphedema, foreign bodies, or previous radiation therapy [1–4]. With a high risk of local recurrence and metastasis, the prognosis for angiosarcomas is poor [5]. Given the association of these aggressive malignancies with radiation therapy, there is an important need to understand the biology of these secondary tumors as radiotherapy becomes increasingly utilized in cancer care.

The diagnostic criteria for radiation-induced sarcomas, established by Cahan et al. and later modified by Arlen et al., include previous history of radiotherapy with a latency period of more than 3-4 years, development of sarcoma within a previously irradiated field or in the tissues adjacent to the field, and histologic confirmation [6, 7]. Radiation-associated angiosarcomas (RAA) were first clinically reported by Calnan and Cowdell in 1959 in a patient who developed an abdominal wall angiosarcoma six years after radiotherapy for penile cancer. Since then, RAA have become a well-recognized entity. The incidence of RAA has been rising as the number of patients treated with radiation increases, particularly with the use of adjuvant radiation in breast conservation therapy [2, 4]. Most reported RAA occur in the breast, but RAA can occur at any site of previous irradiation. Latency periods for the development of RAA have been reported up to as long as over 25 years [2, 4]. Often, diagnosis is delayed because the lesions are initially confused with other conditions such as ecchymosis or infection [5, 8]. 

 It is unclear why some patients develop RAA after irradiation while others do not, and the mechanisms of radiation-associated sarcogenesis remain largely unknown. Furthermore, although RAA show histologic features and poor prognostic behavior similar to sporadic angiosarcomas (SA), it is not known whether there are any specific biologic or clinicopathologic distinctions. Few studies have explored the features and pathogenesis of RAA. In addition, some reports suggest a genetic predisposition or mutational event leading to the development of RAA, such as a mutation in the p53, ATM, or KIT gene [9–11]. In particular, exons 5, 6, 7, and 8 of the p53 gene have been previously shown to contain mutations in postradiation sarcoma, and ATM gene mutations leading to a truncated protein have been implicated in radiation-induced malignancy [10, 12, 13]. However, the limited number of patients in each analysis has made general conclusions difficult; as such, specific features of RAA remain poorly characterized.

To better understand RAA and its relation to SA, selected clinical, pathologic, and biologic features of a series of 48 angiosarcoma patients treated at a single institution (11 RAA, 37 SA) were analyzed in this study. Tumor markers were assessed via immunohistochemical analysis of tissue microarrays and DNA sequencing. 

## 2. Materials and Methods

Forty-eight patients with angiosarcomas (37 SA, 11 RAA) treated at the University of Michigan between 1990 and 2004 were identified by searching the institutional pathology and radiation oncology patient databases. Clinical and followup information were obtained by chart review and the Social Security Death Index (SSDI). Diagnosis of angiosarcoma was verified by pathology review. Additional pathology analysis assessed tumor grade and cytoarchitectural features. Tumors were graded using a 2-tiered system of low and high grades. Architecture was classified as vasoformative, solid, sieve, spindle, epithelioid, or a combination of types. Breast angiosarcomas were characterized as either cutaneous or parenchymal in origin. After pathologic review, a tissue microarray (TMA) was constructed from the most representative areas using the methodology of Nocito et al. [14].

### 2.1. Immunohistochemical Staining

Immunohistochemical staining was performed on a DAKO Autostainer (DAKO, Carpinteria, CA) using DAKO LSAB+ and diaminobenzadine as the chromogen. Deparaffinized sections of the TMA at 5-micron thickness were labeled with antibodies to p53 (rabbit polyclonal antibody, 1 : 100, NCL-p53-CM1 Novocastra, Newcastle, UK), hTERT (mouse monoclonal antibody, 1 : 100, NCL-hTERT Novocastra, Newcastle, UK), and Ki-67 (mouse monoclonal antibody, 1 : 100, MIB-1, DAKO, Carpinteria, CA). p53 and hTERT immunohistochemistry required microwave citric acid epitope retrieval. Staining with Ki-67 required microwave antigen retrieval in high pH buffer. Appropriate negative (no primary antibody) and positive controls were stained in parallel with each set of tumors studied.

TMA cores stained with anti-p53 and anti-hTERT antibodies were scored as positive if strong nuclear staining was identified and negative if no nuclear staining was identified. TMA cores stained with anti-Ki-67 antibody were scored as 0, <10, 10, 25, 50, or >50% based on the percentage of cells demonstrating nuclear staining.

### 2.2. Sequence Analysis of p53 and ATM

DNA was extracted from three 5-micron thick sections of each angiosarcoma block specimen using a Nucleon HT DNA extraction kit (Amersham Biosciences, Piscataway, NJ) according to the manufacturer's instructions. Genomic exons 5, 6, 7, and 8 of the p53 gene and exons 8, 33-34, and 62-63 of the ataxia telangiectasia mutated (ATM) gene were separately amplified according to the methods of De Vos et al. and Liu et al., respectively [15, 16]. These exons were selected for investigation based on published data indicating a role for mutations in exons 5–8 of the p53 gene in postradiation sarcoma, as well as possible involvement of ATM gene mutations which lead to truncation [12, 13]. Amplified product was purified using a Wizard SV PCR clean-up kit (Promega, Madison, WI) and sequenced directly within the University of Michigan Medical Center DNA Sequencing Core using an ABI 377 DNA sequencer (ABI, Foster City, CA). Chromatograms were downloaded directly to CodonCode Aligner software (v.1.5.2, Dedham, MA 02026), and the sequence was compared with the reference sequence downloaded from the National Center for Biotechnology Information (NCBI). The reference sequence numbers for p53 and ATM are NM_000546 and NM_000051, respectively (http://www.ncbi.nlm.nih.gov/).

### 2.3. Statistical Analysis

Categorical and continuous covariates were compared between SA and RAA groups using the Fisher's exact test and *t*-tests, respectively. Survival was estimated using the product-limit method of Kaplan and Meier. Associations between covariates and survival time were also explored using the log-rank test with the magnitude of association quantified by hazard ratios from univariable Cox Proportional Hazards regression models. All statistical analyses were performed using SAS statistical software, version 9.2 (SAS Institute; Cary, NC); *P* values < 0.05 were considered significant.

## 3. Results

### 3.1. Clinical Results

In this series of 48 angiosarcoma patients, 11 (22.9%) had prior radiation, including 7 for breast carcinoma (Table 1). Clinical and radiation data were available for 30/37 (81.1%) SA and 11/11 (100%) RAA cases.

The median age at cancer diagnosis for SA patients was 57.6 years (range 11.7–84.8). SA patients were 54% female and 80% Caucasian. Of the 37 cases, SA sites included breast (5), scalp (14), extremities (4), heart (3), and other (11). Median followup for SA patients from date of diagnosis to date of last appointment or death was 21.0 months (range: 1.1–178.0). SA patients were most commonly treated with a combination of surgery and radiotherapy, but trimodality and single modality therapy, including surgery or chemotherapy alone, were also given.

For RAA patients, the median age at the time of primary cancer diagnosis was 52.7 years (range 16.5–74.3), while the median age at RAA diagnosis was 62.7 years (range 49.1–82.4). RAA patients were 82% female and 100% Caucasian. The primary malignancies of the 11 RAA patients were as follows: breast carcinoma (7), squamous cell carcinoma of the cheek (1), seminoma (1), prostate carcinoma (1), and Hodgkin's lymphoma (1). Median dose delivered to the primary site was 61 Gy (range 52–72.0). Patients were treated with 2D or 3D external beam radiation therapy (EBRT), non-IMRT, using photons and/or electrons. RAA were defined as angiosarcomas that occurred within the prior radiation field. Median and mean latency between radiation and diagnosis of RAA was 7.4 and 11.9 years, respectively (range: 3–35). For management of RAA, all patients received chemotherapy, surgery, additional radiation therapy, or a combination of these modalities at the discretion of the treating physician (Table 1). Of note, all 7 breast RAA patients underwent mastectomy and chemotherapy, but only 1 was treated with additional radiation therapy. Median followup for RAA patients from date of RAA diagnosis to date of last appointment or death was 23.5 months (range 2.8–102). There were no chronic sequelae from radiation therapy, including lymphedema or hyperpigmentation, reported by any patient who developed RAA.

At the time of last followup, for SA patients, there were 28 deaths, with 17 dead of disease (46%). Nine SA patients (24%) remained alive after an average of 54 months following diagnosis: 6 were alive without evidence of disease, 2 were alive with disease, and the disease status of 1 was unknown. For RAA patients, there were 5 deaths (45%): 4 died of disease (36%) and 1 died of unknown causes (9%). Six RAA patients (55%) remained alive after an average of 42 months following diagnosis: 5 were alive without evidence of disease (45%) and the disease status of 1 was unknown (9%). Treatment failure for SA and RAA patients was due to local recurrence and/or development of distant metastases. Median survival from time of angiosarcoma diagnosis to death for SA patients was 21.1 months (95% CI 10.4–70.2) compared to 39.2 months (95% CI 8.0–inf.) for RAA patients, but this difference was not statistically significant (*P* = 0.82).

Analysis of RAA cases by location revealed a difference in survival by breast versus nonbreast sites. Notably, 4/4 (100%) patients with nonbreast RAA died; 3 (75%) patients died of disease with a median overall survival of 13.0 months (95% CI 10.3–39.2). In comparison, only 1/7 (14%) breast RAA patients died, with a median overall survival time that is yet to be reached (*P* = 0.03, Figure 7). At last followup, 5/7 (71%) breast RAA patients had no evidence of disease. The mean latency for breast RAA cases (*n* = 7) after radiation was 6.7 years, compared to 20.9 years (*P* = 0.15) for the nonbreast RAA cases (*n* = 4). Age at the time of angiosarcoma diagnosis was similar for patients with breast versus nonbreast sites (mean 64.9 versus 66.8 years old, resp., *P* = 0.82). There was a trend toward an increased risk of death for patients with a nonbreast RAA site (HR = 8.1, 95% CI 0.8–78.3, *P* = 0.07).

### 3.2. Histopathologic Results

#### 3.2.1. Pathology

**Figure 1:**
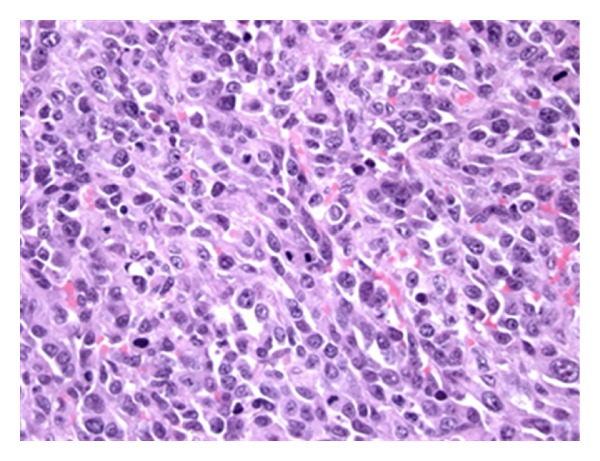
Sheet-like growth pattern, high-grade cytological atypia, and numerous mitotic figures are depicted in this radiation-associated angiosarcoma of the oral cavity. 7/9 cases (77.8%) of radiation-associated tumors in this study were high grade (H&E, 400x).

**Figure 2:**
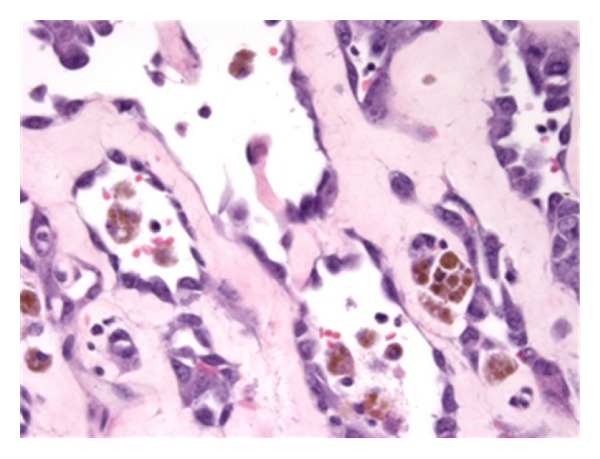
Prominent vasoformative architecture, as depicted in this radiation-associated angiosarcoma of the breast, was seen in the majority of the radiation-associated tumors in this study (H&E, 400x).

**Figure 3:**
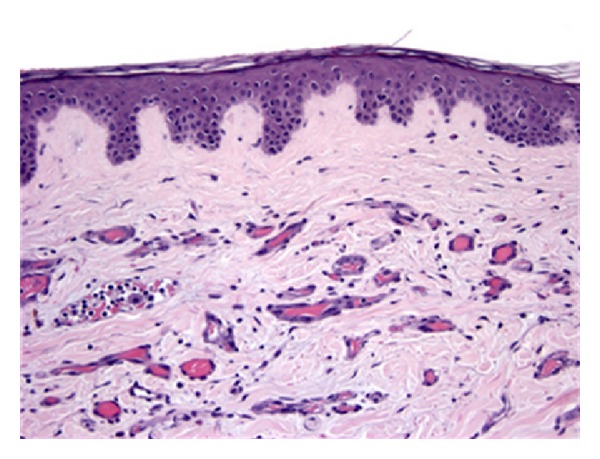
In the breast, most cases of radiation-associated angiosarcoma were cutaneous tumors, evidenced by extensive dermal infiltration in this case (H&E, 200x).

**Figure 4:**
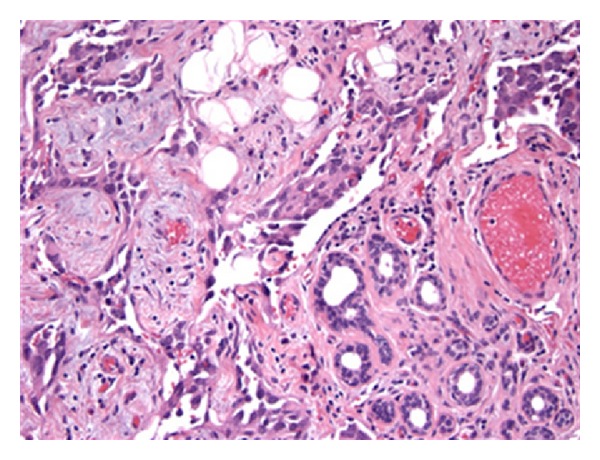
Unlike radiation-associated tumors, the majority of sporadic angiosarcomas of the breast were parenchymal in origin. This micrograph depicts infiltration and entrapment of lobular acini (lower right) by vasoformative angiosarcoma (H&E, 200x).

Pathological material was available for 37/37 (100%) SA and 9/11 (82%) RAA cases (Table 2). There were no histopathological features that distinguished RAA from SA (Figures 1 and 2). Both tumors were classified as high grade (SA 86.5%, RAA 77.8%; *P* = 0.609) and characterized by a variety of architectural patterns, although RAA tended to have well-defined vasoformative areas (55.6% versus 27%, *P* = 0.127). Among breast angiosarcomas, RAA were predominantly cutaneous in origin (80.0%), while the majority of breast SA were parenchymal (80.0%) (*P* = 0.21) (Figures 3 and 4).

#### 3.2.2. Immunohistochemistry and TP53/ATM Mutational Analysis

**Figure 5:**
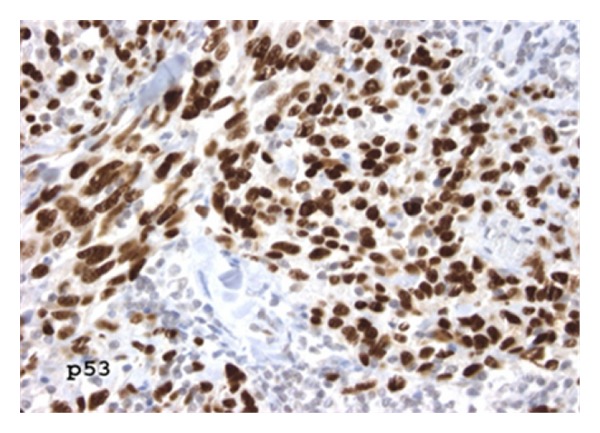
Although significant p53 staining was present in a quarter of the sporadic angiosarcomas as depicted, none of the radiation-associated tumors were positive (immunoperoxidase, 400x).

**Figure 6:**
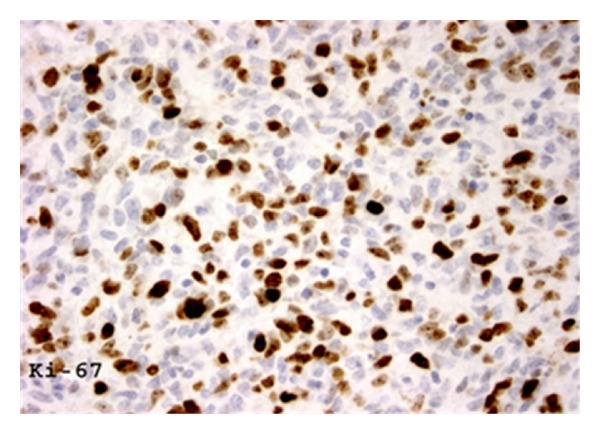
Nuclear staining for Ki-67 in greater than half the cells was present in 40% of radiation-associated angiosarcomas, indicating very high proliferative activity (immunoperoxidase, 400x).

**Figure 7:**
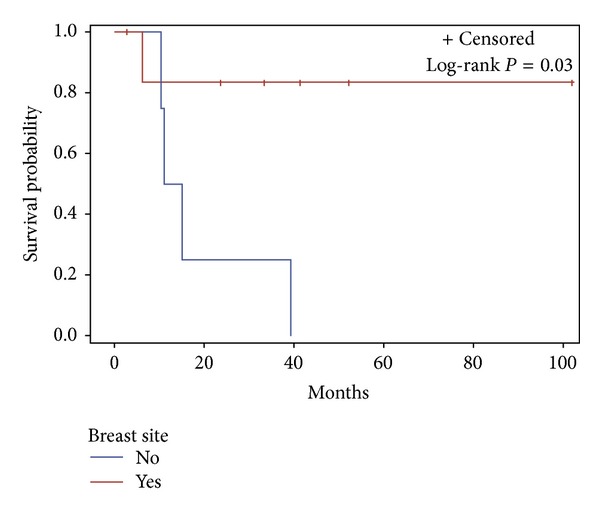
Overall survival in RAA patients with breast site versus nonbreast site.

Overexpression of p53 by immunohistochemistry was limited to SA cases (24.3% SA versus 0% RAA, *P* = 0.21) (Table 3; Figure 5). Proliferative activity was similar in both types of angiosarcomas with ≥50% Ki-67 nuclear staining in 35.3% SA and 44.4% RAA (*P* = 0.25) (Figure 6). hTERT was universally expressed in all SA and RAA cases alike. Mutation of TP53 was identified in 13.5% of SA and 11.1% of RAA (*P* = 1.00). Neither SA nor RAA cases demonstrated ATM mutations.

## 4. Discussion

Angiosarcomas are rare, aggressive tumors that can arise de novo or after radiation therapy. Because of the relative rarity of disease and paucity of cases, there has been little characterization to date comparing the clinicopathologic and biologic associations between sporadic and radiation-associated angiosarcomas. Although infrequent, RAA is a well-recognized complication of radiation therapy, particularly in the treatment of breast cancer. Previous SEER data have reported a 16-fold increase in risk of breast RAA after treatment with radiation for primary breast cancer, while the Dutch have noted a 3,200-fold increase in relative risk [17, 18]. In the literature, the incidence of RAA ranges from 0.05 to 0.16% [10, 19]. As the use of radiation therapy becomes increasingly widespread, a concomitant rise in the incidence of RAA is anticipated. Therefore, it is important to understand the underpinnings of this potential sequela of treatment. In this series of 48 angiosarcoma patients, we sought to characterize RAA from a clinicopathologic and biologic perspective as well as to delineate its relationship to SA.

Clinically, as expected with secondary malignancies, RAA patients were slightly older at presentation than SA patients (median age 62.7 versus 57.6 years) but approximately the same age at primary cancer diagnosis (6th decade). In our series, the median latency between radiation and diagnosis of RAA at all sites was 7.4 years (range 3–35). For breast RAA, the latency period was 7 years, which was the same as that reported by Seinen et al. in their series of 35 breast RAA patients whose demographics were notably similar to ours in terms of age at the time of diagnosis of breast cancer and RAA [20].

 Although the median survival after time of angiosarcoma diagnosis was shorter for SA versus RAA patients (21.1 versus 39.2 months, resp.), the overall survival distributions were not statistically different (*P* = 0.82). The relatively poor survival experience of our series is consistent with other published studies. The median survival for RAA patients varies slightly from series to series, ranging from 14.5–34 months as reported by Monroe, 18–30 months by Tahir, and 12–60 months by Monroe et al., Tahir et al., and De Bree et al. [2, 8, 10]. Hodgson and Vorburger found no difference in survival between SA and RAA of the breast [3, 21]. Hodgson reported an RAA mortality rate of 44–58% [21]. Our data, in agreement with conclusions from other case series, suggest that the natural history of RAA and SA is similar.

With regard to breast angiosarcomas, most other studies demonstrate that RAA are associated with better outcomes compared to SA. In contrast, in a series of 28 consecutive cases of breast angiosarcoma (8 SA, 20 RAA) diagnosed from 1999 to 2009 at the European Institute of Oncology, Fraga-Guedes and colleagues reported a poorer prognosis in RAA versus SA (5 year OS 28.2% versus 85.7%, *P* = 0.066) [22]. 

Unlike most previous studies, we also attempted to characterize RAA occurring in different sites, rather than limiting cases to the breast. We noted that SA most commonly involved the scalp (14/37, 37.8%), whereas RAA primarily developed in the breast (7/11, 63.6%) (Table 1). The relatively high incidence of RAA in the breast may in part be related to length of patient survival after radiation therapy but may also indicate an underlying cellular difference in breast tissue, making it more susceptible to radiation damage. Indeed, this observation is supported by several studies which report RAA development after breast-conserving therapy [2, 3, 8, 9, 21]. Given a higher incidence of breast RAA in relation to other RAA sites, the characterization of breast RAA and SA is particularly important as a higher proportion of patients are affected.

Furthermore, our clinical findings demonstrate a shorter mean latency period of 6.7 years for breast RAA compared to 20.9 years for nonbreast RAA (*P* = 0.15). This finding is consistent with observations in other studies; Billings et al. found in their series that breast RAA had a median latency of 59 months, which was half that of Stewart-Treves angiosarcoma (STAS) and much shorter compared to the latency of RAA at other sites, which frequently exceeds 10 years [9]. Others have also reported a shorter latency period for breast RAA, most commonly ranging from 1 to 7 years [2, 3, 10, 23], compared to nonbreast RAA, ranging from 10 to 30 years [1, 3, 24].

Analyses reveal that 4/4 (100%) patients in our series with nonbreast RAA died of disease, while only 1/7 (14%) patients with breast RAA died of disease. The median survival for nonbreast RAA was markedly lower than for breast RAA (13.0 months versus yet to be reached, log-rank *P* = 0.03) after a median followup of 23.5 months. This observation correlates with an increased risk of death for nonbreast RAA compared to breast RAA (HR = 8.1, CI 0.8–78.3, *P* = 0.07). Although the patient population is small, the gaps in survival and hazard ratio for death between breast and nonbreast RAA demonstrate a compelling contrast in the behavior of RAA based on site.

Our data supports a distinct difference between breast RAA and nonbreast RAA in disease latency and survival. This suggests that other factors may be involved in development of RAA, such as edema, chemotherapy, volume of tissue irradiated, radiation dose heterogeneity, or simply inherent properties of the breast tissue itself [23, 24]. Indeed, a study by Sheppard found that the use of chemotherapy during radiation treatment increased the relative risk of sarcoma development by a factor of 4.7–9.0 [25]. Furthermore, radiotherapy for breast cancer is uniquely associated with breast edema, whole breast and skin irradiation, and field edge dose heterogeneity in traditional tangential fields, and any one of these factors may contribute to the differences seen between RAA and nonbreast RAA.

From a histologic standpoint in our series, RAA are indistinct from SA. Both SA and RAA are most often high-grade tumors. The pattern of growth in RAA in our series was predominantly of vasoformative architecture (60%), whereas SA architecture was nonspecific, including vasoformative (27%), solid (21.6%), and a combination of patterns. While differences in architecture did not reach the level of statistical significance, vasoformative architecture has been associated with statistically improved survival in two recent studies by Shon et al. [26] and Deyrup et al. [27]. In our breast angiosarcoma cases, RAA were more often cutaneous in origin (80%), while SA were more often parenchymal in origin (80%). These findings, consistent with data from previous studies, support the dermal origin of breast RAA compared to the parenchymal origin of breast SA [4, 8, 24]. Gladdy et al. examined the prognostic significance of histologic type in radiation-associated soft tissue sarcomas [28]. They found that angiosarcomas comprised 21% of primary radiation-associated sarcomas and that radiation-associated angiosarcomas had decreased survival in comparison to radiation-associated leiomyosarcomas, fibrosarcomas, and myxofibrosarcomas. After adjusting for histologic type, age, tumor size, depth, and margin status, radiation-associated sarcomas were associated with a 1.7-fold worse disease-specific survival compared with sporadic soft-tissue sarcomas.

In our immunohistochemical analyses, less than 50% of RAA demonstrated high Ki-67 expression, while p53 overexpression was entirely absent. hTERT was uniformly expressed. High expression of Ki-67 has been correlated with increased rates of metastasis and mortality in high-grade soft tissue sarcomas. While Ki-67 overexpression has been previously studied as a prognostic indicator of outcomes, the literature uses a variety of definitions for high proliferative index; in this analysis 50% was used as the threshold. On mutational analysis, TP53 mutation was low (10.0%), and ATM mutation was not present. The relative absence of p53 and ATM mutations in RAA was also noted in a case report by De Bree et al. [10]. Based on these results, mutations of p53 or ATM do not appear to drive development of RAA. 

Overall, RAA shared a similar immunohistochemical and mutational profile with SA, except that SA demonstrated p53 overexpression in some cases. Although p53 expression in RAA versus SA cases did not reach statistical significance, the trend toward a difference in expression may indicate a difference in mechanism for tumorigenesis, such that the p53 pathway may not play a role in RAA development. Analyses of a larger number of cases may help elucidate this difference.

 With increasing numbers of patients treated with radiation therapy, the incidence of RAA is likely to continue to rise. Unfortunately, treatment options are relatively limited, although reirradiation is sometimes employed. In the Seinen series, patients were primarily treated with surgical resection, and R0 resection was achieved in 23 of 31 patients. However, local recurrence occurred in 19 patients after a median of only 6 months, which was attributed to the multifocal growth pattern of angiosarcoma, and median disease-specific survival was 37 months [20]. Given the poor local control with surgery alone, Scott et al. reported the University of Florida experience of using hyperfractionated radiation therapy with hyperthermia for treatment of 41 angiosarcoma patients, including 16 RAA cases, and involving a variety of sites. In this series, aggressive treatment with resection and hyperfractionated radiotherapy was associated with the best prognosis [29]. Various taxane-based chemotherapeutic regimens have also shown some benefit, both in the neoadjuvant and unresectable setting. For radiation-associated angiosarcomas in the primary setting, use of docetaxel or paclitaxel has yielded significant response rates [30, 31]. Similarly, recent phase II trials including the ANGIOTAX study have demonstrated a clinical benefit of paclitaxel for unresectable angiosarcoma [32]. Still, despite some advances in treatment options, the prognosis of RAA remains poor. Thus, the implications for characterization of RAA and their relation to SA are important, as these data may not only improve treatment outcomes for RAA and SA but also help prevent the development of RAA. 

In our series, examining histology and several classic immunohistologic markers of aggressive behavior did not help distinguish between SA and RAA. However, as is typical for the study of rare disease, our study was limited due to small patient numbers. With the development of collaborative databases in the future, more extensive tumor characterization in conjunction with patient profiling may enable clinicians to develop predictive models to individualize treatment of RAA, as well as SA. Further studies focusing on gene expression profiling may provide more insight into the biology of RAA and help predict which patients may be at risk of developing RAA. Such predictive models would also allow clinicians to predict which patients would be at risk for developing RAA, thus recommending a treatment course that avoids radiation, such as choosing mastectomy over breast-conserving therapy for selected breast cancer cases. Furthermore, unique properties of each angiosarcoma subtype can be utilized to develop targeted therapies. For example, in vasoformative types of tumors typical of RAA, antivascular targeting agents including bevacizumab may prove effective and are currently in phase II clinical trials. In this way, clinicopathologic and biologic distinctions between breast RAA, nonbreast RAA, and SA may help optimize treatment and prevent development of these aggressive tumors.

## Figures and Tables

**Table 1:** Summary of clinical features in radiation-associated angiosarcoma (RAA) patients.

Case no.	Sex	Primary Dx	Age 1° Dx	Rx	RT dose (Gy)	Interval to RAA (mos)	RAA Tx	Status	Interval to death or last f/u (mos)
1	F	L breast CA	50	L/RT/Raloxifene	60	37	Gem, taxotere/M	DOD	6.1
2	F	L breast CA	57	L/RT	64.6	89	Adria, ifos/M (bil)	A-NED	41.3
3	F	L breast CA	70	L/RT	60	63	M/taxol	A-NED	33.2
4	F	R breast CA	50	L/RT	61	57	Adria, ifos/M	A-NED	102.0
5	F	R breast CA	74	L/RT/Tm	52	84	Vinb, actin-D/M	A-NED	52.2
6	F	R breast CA	53	L/C/RT/Tm	64.6	93	M (bil)/Adria, ifos	A-NED	23.5
7	F	L breast CA	51	L/C/RT	54	141	M/Adria, ifos/RT/Adria, ifos	A	2.8
8	F	SCC, R Cheek	68	WLE/RT	70.2	78	WLE/RT (palliative)	DOD	11.0
9	M	Seminoma	26	WLE/RT	Co × 6 wks	419	Ifos/WLE/RT (63 Gy)	DOD	39.2
10	M	Prostate CA	72	WLE/RT	72	120	WLE	D	10.2
11	F	Hodgkin's lymphoma	16	C/total nodal RT	NA	387	Adria, ifos/amputation	DOD	15.1

CA: carcinoma, SCC: squamous cell carcinoma, L: lumpectomy, RT: radiation therapy, C: chemotherapy, Gem: gemcitabine, Adria: adriamycin, Ifos: ifosfamide, Tm: tamoxifen, M: mastectomy, WLE: wide local excision, D: dead, DOD: dead of disease, A: alive, A-NED: alive-no evidence of disease.

**Table 2:** Histological comparison between SA and RAA.

	Grade	Architecture	Depth of involvement (breast only)
SA (*n* = 37)	High: 32/37 (86.5%)	V: 10/37 (27%) So: 8/37 (21.6%) Si: 5/37 (13.5%) S/E: 4/37 (10.8%) Other: 10/37 (27%)	Dermis: 1/5 (20%) Parenchyma: 4/5 (80%)

RAA (*n* = 9)	High: 7/9 (77.8%)	V: 5/9 (55.6%) So: 1/9 (11.1%) S/E: 3/9 (33.3%)	Dermis: 4/5 (80%) Parenchyma: 1/5 (20%)

V: vasoformative, So: solid, Si: sieve, S/E: solid/epithelioid.

**Table 3:** Immunohistochemical and mutational analysis of prognostic markers comparing sporadic angiosarcomas (SA) and radiation-associated angiosarcomas (RAA).

	p53 overexpression	Ki-67		hTERT	TP53 mutation	ATM mutation
		<50%	≥50%			
SA	9/35 (25.7%)	22/34 (64.7%)	12/34 (35.3%)	37/37 (100%)	5/37 (13.5%)	0/37 (0%)

RAA	0/9 (0%)	5/9 (55.6%)	4/9 (44.4%)	9/9 (100%)	1/9 (11.1%)	0/9 (0%)
